# Risk scores for predicting incidence of type 2 diabetes in the Chinese population: the Kailuan prospective study

**DOI:** 10.1038/srep26548

**Published:** 2016-05-25

**Authors:** Anxin Wang, Guojuan Chen, Zhaoping Su, Xiaoxue Liu, Xiangtong Liu, Haibin Li, Yanxia Luo, Lixin Tao, Jin Guo, Long Liu, Shuohua Chen, Shouling Wu, Xiuhua Guo

**Affiliations:** 1 Department of Epidemiology and Health Statistics, School of Public Health, Capital Medical University, Beijing, China; 2 Beijing Municipal Key Laboratory of Clinical Epidemiology, Capital Medical University, Beijing, China; 3 Department of Neurology, Tangshan Gongren Hospital, North China University of Science and Technology, Tangshan, China; 4 Department of Epidemiology and Health Statistics, Academy of Public Health and Management, Weifang Medical University, Weifang, China; 5 Department of Cardiology, Tangshan People’s Hospital, Tangshan, China; 6 Department of Cardiology, Kailuan Hospital, North China University of Science and Technology, Tangshan, China

## Abstract

Few risk scores have been specifically developed to identify individuals at high risk of type 2 diabetes in China. In the present study, we aimed to develop such risk scores, based on simple clinical variables. We studied a population-based cohort of 73,987 adults, aged 18 years and over. After 5.35 ± 1.59 years of follow-up, 4,726 participants (9.58%) in the exploration cohort developed type 2 diabetes and 2,327 participants (9.44%) in the validation cohort developed type 2 diabetes. Age, gender, body mass index, family history of diabetes, education, blood pressure, and resting heart rate were selected to form the concise score with an area under the receiver operating characteristic curve (AUC) of 0.67. The variables in the concise score combined with fasting plasma glucose (FPG), and triglyceride (TG) or use of lipid-lowering drugs constituted the accurate score with an AUC value of 0.77. The utility of the two scores was confirmed in the validation cohort with AUCs of 0.66 and 0.77, respectively. In summary, the concise score, based on non-laboratory variables, could be used to identify individuals at high risk of developing diabetes within Chinese population; the accurate score, which also uses FPG and TG data, is better at identifying such individuals.

According to the second *Global Status Report on Noncommunicable Diseases 2014* released by the World Health Organization, the prevalence of diabetes in adults aged 18 years and over in China is estimated to be 9.5%^1^. The actual number of patients with diabetes in China is therefore a shocking figure because of the country’s large population. The financial costs of diabetes-related health expenditures also weigh heavily on the nation’s economy. Thus, reducing diabetes-related morbidity in China is an important issue.

Type 2 diabetes can be prevented and delayed in high-risk individuals by modifying their lifestyle^2^,^3^. However, the inconvenience and relatively high cost of the 2-hour oral glucose tolerance test (OGTT), which is generally used to identify high-risk subjects^4^, limits its use. On the other hand, the application of risk assessment tools based on demographic, anthropometric, and simple laboratory tests to screen high-risk subjects is both feasible and economical^5^,^6^,^7^.

A number of risk assessment tools based on readily available clinical variables have been developed to predict the incidence of new diabetes cases^4^,^6^,^7^,^8^,^9^,^10^,^11^,^12^,^13^,^14^. These are derived from European^6^,^10^,^15^, American^4^,^7^,^8^,^13^,^15^, Australian^14^, and Asian^9^,^11^,^12^ studies. However, differences in ethnicities, territories, and lifestyles may limit the application of some of these effective risk scores to the Chinese population. Developing a risk score to specifically identify subjects at high risk of type 2 diabetes that may be widely used in China is therefore both urgent and necessary.

Few risk scores have been developed specifically to identify individuals at high risk of type 2 diabetes in China^11^,^16^; most have been generated from cross-sectional data, and are limited by small sample size. In the present study, we aimed to develop and validate simple risk scores based on self-assessed information and simple laboratory measurements in the Kailuan study characterizing individuals at increased risk of developing type 2 diabetes during a follow-up period of nearly 5.35 years. Furthermore, we compared the performance of other algorithms using our cohort^4^,^6^,^7^,^8^,^9^,^10^,^11^,^12^,^13^,^14^,^17^,^18^,^19^.

## Results

### Baseline characteristics and follow-up

Our study included 73,987 participants with a mean age of 49.76 ± 12.04 years. Of these, 58,329 (78.8%) were men, and 68,349 (92.4%) had an education level of high school or below. The average body mass index (BMI) was 24.96 ± 3.46 kg/m^2^, and the average fasting plasma glucose (FPG) level was 5.09 ± 0.68 mmol/L.

Of the 49,325 participants without diabetes at the baseline examination in the exploration cohort, 4,726 (9.58%) developed diabetes during the mean 5.35 year follow-up period. Among these 4,726 participants who developed diabetes, 3,745 were diagnosed via FPG determination, 319 were diagnosed through their self-reported history of diabetes or use of anti-diabetic medicine, and 662 were diagnosed using two or three criteria. Of the 24,662 participants in the validation cohort, 2,327 (9.44%) developed diabetes.

The baseline characteristics of the exploration and validation cohorts are shown in Table 1. The values of all variables determined for the validation cohort differed from those of the exploration cohort (all *P* > 0.05). Participants who developed new-onset diabetes after 5.35 years in both cohorts were more likely to be male, married, older, and heavier and to have a family history of diabetes, cerebrovascular diseases, drinking and smoking habits, and higher waist circumference, waist-to-hip ratio, triglyceride (TG), total cholesterol (TC), FPG, resting heart rate, blood pressure (BP), and BMI. They also had lower education levels and lower income levels than those who did not develop diabetes. There were significant differences between newly diabetic and non-diabetic participants in the exploration cohort, in all variables except for high density lipoprotein (HDL), sleep duration and salt intake at a *P *< 0.05 threshold. However, all the candidate variables except for sleep duration met the model entry criteria at the *P *< 0.2 level.

### Exploration and validation of prediction scores

**Figure 1:**
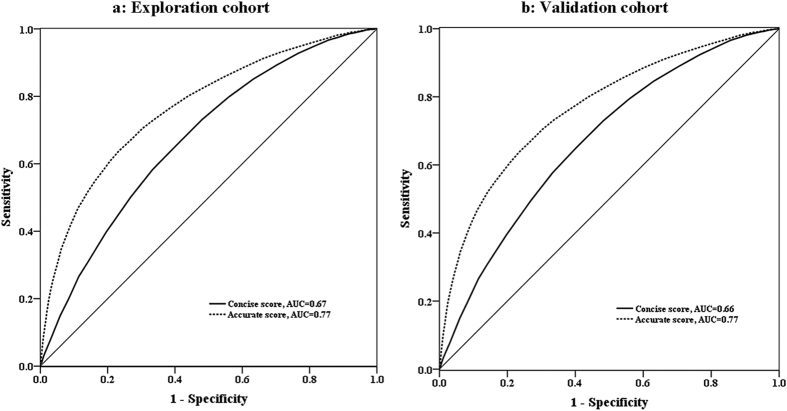
Receiver operating characteristic curves for the concise score and accurate score in the exploration cohort and validation cohort. Key: AUC – area under the receiver operating characteristic curve.

Table 2 shows the risk scores derived from the exploration cohort using a Cox proportional hazards model. Of the 24 variables that initially entered into the model, only age, gender, BMI, family history of diabetes, education, BP, resting heart rate, FPG, and TG or using lipid-lowering drugs made significant contributions to the score. These statistically significant risk factors constitute the accurate score. Excluding FPG and TG or using lipid-lowering drugs produces the concise score. People with FPG values ≥ 6.1 mmol/L had the highest risk score for predicting incidence of diabetes. The receiver operating characteristic (ROC) curves (Fig. 1a) demonstrate that the accurate score has a better predictive capacity than the concise score (areas under the curve (AUC) of 0.77 and 0.67, respectively, *P *< 0.001). The total scores for the concise score varied from 0–37, while the accurate score ranged from 0–60.

The diagnostic characteristics of the two models using the validation cohort are shown in Table 3. The concise score had a performance corresponding to an AUC of 0.66 (95% confidence interval (CI): 0.65–0.68); the AUC for the accurate score was 0.77 (95% CI: 0.76–0.78) (Fig. 1b). The concise score exhibits a reasonable sensitivity of 0.72 and specificity of 0.52, with an optimum cut-off value of 21. The accurate score exhibits a reasonable sensitivity (0.70), and specificity (0.70) with an optimum cut-off value of 27. Stratified analyses show that the concise score performs better in women than in men (AUC: 0.72 vs. 0.65), as does the accurate score (AUC: 0.81 vs. 0.76), and the concise score performs better for people <60 years compared to those ≥60 years (AUCs of 0.67 and 0.62, respectively), but the accurate score performs slightly better for people ≥60 years (AUC: 0.76 vs. 0.77).

**Figure 2:**
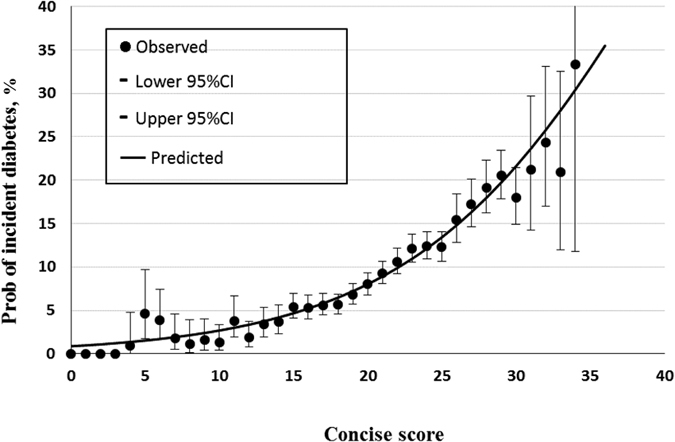
Calibration plot for the concise score using the validation cohort. The dots represent the observed rates of incident diabetes, and the vertical lines represent the 95% confidence intervals. The continuous line represents the predicted probability of incident diabetes.

**Figure 3:**
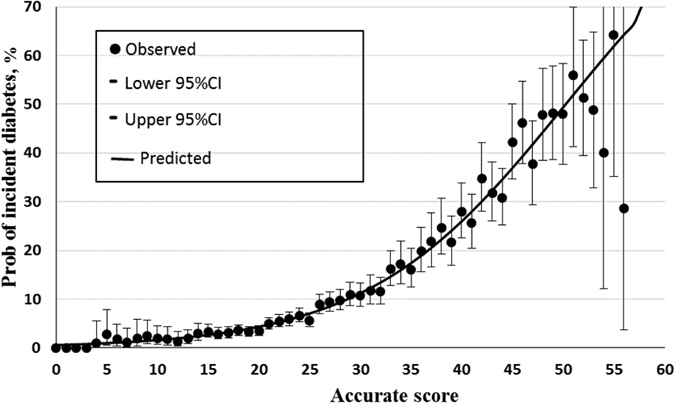
Calibration plot for the accurate score using the validation cohort. The dots represent the observed rates of incident diabetes, and the vertical lines represent the 95% confidence intervals. The continuous line represents the predicted probability of incident diabetes.

Figures 2 and 3 present the calibration plots for the concise and accurate scores using the validation cohort, with the probability of incident diabetes after a mean of 5.35 years on the ordinate, and scores on the abscissa. The dots represent the actual incidence of diabetes, and the vertical lines represent the 95% CIs. The continuous line represents the predicted probability of incident diabetes, which clearly increases with increasing score. At the cut-off value of 21 in the concise score the predicted probability is 9.29%; the corresponding value for the accurate score (at 27) is 9.42%.

### Validation of previous scores

Table 4 summarizes the performance of 14 other diabetes risk scores. These include 7 scores containing laboratory variables^4^,^7^,^8^,^11^,^12^,^13^,^19^ and 7 scores without laboratory data^6^,^9^,^10^,^14^,^15^,^17^,^18^. When applied to the validation cohort (1/3 of the whole cohort), none of the 7 scores containing laboratory variables outperformed our accurate score. Our concise score also performs better than the other 7 scores that do not contain laboratory variables. Both of our scores (concise and accurate) outperform the New Chinese Diabetes Risk Score devised by Zhou *et al*.^17^ (AUCs of 0.66 and 0.77 vs. 0.61) when used to predict the incidence of diabetes in individuals. When applied to the whole sample, the diabetes risk score developed by Schmidt *et al*.^8^ performs the best out of the 14 risk scores (AUC of 0.74).

## Discussion

Using two-thirds of the Kailuan cohort, we derived two scoring systems to predict the incidence of diabetes among Chinese adults after a mean follow-up period of 5.35 years. We validated both of the scores using the remaining one-third and confirmed their predictive capacity for incident diabetes. The concise score is non-invasive and can be performed by the individuals themselves. The accurate score is more effective in predicting diabetes but requires simple blood tests. Our scores performed better than the 14 scores derived from other populations.

The AUCs for 14 previous diabetes risk scores ranged from 0.62 to 0.87 in their original populations^4^,^6^,^7^,^8^,^9^,^10^,^11^,^12^,^13^,^14^,^17^,^18^,^19^, and ranged from 0.52 to 0.73 in the current study population. Our accurate score performed with a moderately high AUC value (0.77) and our concise score performed with a somewhat low AUC value (0.67). Of note, a model providing an AUC value < 0.80 for predicting incident diabetes may be limited in its clinical utility. However, all predictors included in our scores are readily available clinical variables. If further predictors related to blood testing were included, the scores would perform better.

In our scores, the FPG variable is the strongest predictor of incident diabetes (a contribution of up to 20 points). This result is consistent with previous reports^7^,^8^,^11^,^13^,^19^. Impaired fasting glucose (IFG) has been defined at the levels from 6.1 to 6.9 mmol/L^20^,^21^, and from 5.6 to 6.9 mmol/L^22^. It is not surprising that individuals with IFG have a high risk of developing diabetes. In the accurate score, we also found that the points contributed by the category from 6.1 to 6.9 mmol/L was about twice that of the points contributed by the category from 5.6 to 6.1 mmol/L. The risk of incident diabetes increased with the high FPG level^8^.

Age was the second-strongest predictor in our scores; indeed, it has been included in most of the published scores used to predict incident diabetes^4^,^6^,^7^,^8^,^9^,^10^,^11^,^12^,^13^,^14^,^17^,^18^,^19^,^23^,^24^. Individuals aged ≥60 years have the highest risk of developing diabetes in our scores (accounting for 29.7% of the total score in the concise score), closely followed by individuals in the age range from 40 to 59 years. In contrast, in the simple score used by Aekplakorn *et al*.^9^, the category ≥50 years was considered to have the highest point contribution (accounting for 11.8% of the total score). Although they differ in the age cut-off value, these scores are consistent in that older age predicts incident diabetes. In addition, some scores that were developed in a particular age group^6^,^12^,^13^,^19^, included age as a continuous variable^4^,^8^,^9^,^10^,^11^,^18^,^14^,^17^, and also suggest that the risk of incident diabetes increases with older age.

In our concise score, BMI was the second-strongest predictor after age. In previous diabetes risk scores, BMI or waist circumference were also strong predictors^4^,^6^,^7^,^8^,^9^,^10^,^11^,^12^,^13^,^14^,^17^,^18^,^19^,^23^,^24^. Compared to height, the variables of weight, waist circumference, and BMI had more statistical significance in the univariate analysis. When all of these factors were entered into the Cox proportional hazards score, only the BMI made a contribution to the scoring system. Similarly, in the clinical diabetes risk scores by Balkau *et al*.^15^, both BMI and waist circumference had similar predictive value, but only waist circumference was included in the score. The variables of BMI and waist circumference may not coexist in the same scoring systems. In addition, BMI was not recommended as a candidate variable in the report by Kahn *et al*.^13^, because that BMI is a complex index; thus, there is a possibility that the association between BMI and incident diabetes might be driven as much by reduced height as by increased weight.

We are not the first researchers to include resting heart rate in a diabetes prediction score^13^. Both the basic and enhanced scores developed by Kahn *et al*.^13^ included the resting heart rate, and were assigned points of 2 and 5 with mean scores of 38.1 and 33.7, respectively. European studies^25^,^26^ and a Chinese study from the Kailuan database^27^ also demonstrated that an elevated resting heart rate is an independent risk factor for incident diabetes. It has been proposed that sympathetic activation resulting in increased heart rate may lead to insulin resistance which increases diabetes risk^28^. Ultimately, the exact mechanism for this remains to be elucidated.

The inclusion of TG and BP in the diabetes risk score is also not new^7^,^8^,^11^,^12^,^13^,^19^. A widely held viewpoint is that the incidence of type 2 diabetes is the result of complex metabolic processes^29^,^30^. Elsewhere, it has been demonstrated that high normal BP and hypertension are associated with an increased risk of developing type 2 diabetes^31^. As reported in previous studies^32^,^33^, higher TG and lower HDL levels are also associated with incident diabetes. However, only TG made a contribution to incident diabetes in our accurate score, which is consistent with the scores by Kanaya *et al*.^19^ and Gao *et al*.^12^. The diabetes risk scores developed by Schmidt *et al*.^8^, Wilson *et al*.^7^, Chien *et al*.^11^, Meigs *et al*.^24^, and Kahn *et al*.^13^, included both the TG and HDL variables, while the score by Stern *et al*.^4^ only included the HDL variable.

A family history of diabetes is also an important predictor for incident diabetes; genetic and environmental pathways may account for this^24^. ‘Current heavy smoker’ was given the highest point value in the German Diabetes Risk Score^10^. In the clinical scores by Balkau *et al*.^15^, smoking was the second most important predictive factor for men, but was not a predictor for women. However, smoking did not contribute to any of our scores, which was consistent with the majority of previously developed diabetes risk scores^4^,^6^,^7^,^8^,^11^,^13^,^19^. Physical activity frequency was also not predictive, possibly because of its negative correlation with BMI.

There were some limitations to our study. First, the study is based on residents in the Kailuan community of Tangshan, which might not be representative of the general population of China. In particular, the Kailuan study population is exposed to environmental pollution, and a large proportion of the participants were manual workers, including coalminers. Furthermore, the average BMI of participants included in the current study is higher than the national average^34^. The two scoring systems we developed will need to be validated in other parts of China or in other countries. Second, our scores were derived and validated using the same cohort. This may reduce their ability to predict incident diabetes in other populations. We hope to test these scores in other population samples in the future. Third, we did not collect further parameters related to blood testing. One-hour plasma glucose has been demonstrated as a strong predictor of incident diabetes^35^, and single-nucleotide polymorphisms are known to have associations with the risk of diabetes^24^. These, if included, may have improved the discrimination of the accurate score. Another limitation is that we have not been able to include OGTT data in our diagnostic criteria. This is likely to have led to an underestimate of the association between diabetes and score parameters. On the other hand, our scores used parameters that are easy to obtain, and are appropriate in China.

## Conclusion

We designed two scores for use as assessment tools to identify subjects at high risk of developing type 2 diabetes among the Chinese population. The concise score is non-invasive and can be used by the individuals themselves. The accurate score provides superior assessment ability but requires simple blood tests. Our scores performed better than other existing diabetes risk scores within the Chinese study population. Further research is required to test the scores we developed in other population samples of China.

## Methods

### The Kailuan study

From June 2006 to October 2007, a population-based cohort of 101,510 people (81,110 males and 20,400 females, 18–98 years old) were recruited for the Kailuan study^36^,^37^. The participants consisted of on-job and retired workers in the service of the Kailuan Coal Mine Group Corporation and residing in the Kailuan community. They were recruited from 11 hospitals responsible for the health care of the community^38^. The community is located in the Tangshan area of northern China. Periodic health examinations, including questionnaire interviews, anthropometric measurements, clinical examinations, and laboratory assessments, were performed in 2-year cycles until the present day. We used the data for the period from 2006 to 2012 in this study.

Individuals were eligible for enrolment if they were aged 18 years or over, provided informed consent, and updated their health status every 2 years according to the protocol. In the present study, 9,268 participants were excluded due to missing information related to candidate variables, and 8,766 were excluded due to missing follow-up data. Another 9,489 participants were excluded because they had either a baseline FPG level higher than 7.0 mmol/L (≥126 mg/dL), or a history of diabetes (as informed by a physician), or used anti-diabetic medicine. The remaining 73,987 individuals were available for our analyses.

The study followed the guidelines of the Helsinki Declaration, and was approved by the Ethics Committees of both the Kailuan General and Beijing Tiantan hospitals. All participants provided their written informed consent.

### Assessment of risk factors and outcomes

The candidate baseline variables presented in Table 1 were chosen for their common availability and use in previous diabetes risk scores. The demographic data and information about lifestyle characteristics, medication use, history of diseases, and family history were obtained using questionnaires that were administered by research doctors of the hospitals who were specially trained for the task. The classification of each category variable has been described elsewhere in some detail^38^,^39^. To further clarify, the physical activity group ‘very active’ was defined as more than 80 minutes of activity per week, ‘moderately active’ corresponded to less than 80 minutes per week, and ‘inactive’ meant no physical activity. The salt intake group ‘high’ was defined as 10 grams/day, ‘medium’ as 6–10 grams/day, and ‘low’ as 6 grams/day. The ‘smoking occasionally’ group is defined as one cigarette or less per day and ‘smoking frequently’ as smoking daily. The ‘drinking occasionally’ group was defined as drinking 1–3 times every month, while ‘drinking frequently’ was defined as drinking daily.

Height, weight, hip circumference, and waist circumference (2.5 cm above the umbilicus) were measured in the standing position, without heavy clothing, to the nearest 0.1 cm or 0.1 kg by nurses responsible for annual routine health examinations. Waist-to-hip ratio was calculated as waist circumference divided by hip circumference. The waist measurement was categorized based on the dividing points of 84 and 90 for men and 77 and 84 for women, in reference to Korean diabetes risk scores in which the population had a similar Asian nature^40^. BMI was calculated according to the equation BMI*** *****= **weight (kg)/height (m)^2^ and was classified based on the common Chinese criteria, i.e., normal corresponds to BMI < 24.0 kg/m^2^, overweight to 24.0 ≤ BMI < 28.0, and obese to BMI ≥ 28.0. Two measurements of BP were taken with a 5-minute interval. If the two measurements differed by more than 5 mmHg, then an additional reading was taken, and the final, average of the readings used for analysis purposes. The resting heart rate^27^ was based on the results of a 12-lead electrocardiogram performed with the participants in the supine position.

Blood samples were collected in the morning after an overnight fast in the 11 hospitals and analysed at the central laboratory of the Kailuan General Hospital. FPG was measured using the hexokinase/glucose-6-phosphate dehydrogenase method. TG, TC, HDL, and low density lipoprotein (LDL) levels were all measured enzymatically. According to the criteria of the *National Cholesterol Education Program Adult Treatment Panel III*^41^, a TG level of 1.70 mmol/L (150 mg/dL) or greater is considered to be hypertriglyceridemia. Similarly, an HDL level less than 1.03 mmol/L (40 mg/dL) in men, or 1.29 mmol/L (50 mg/dL) in women, was considered low. A TC level of 5.18 mmol/L (200 mg/dL), and LDL level of 3.35 mmol/L (130 mg/dL), were considered borderline-high levels.

The outcome of interest in the present study is the first incidence of diabetes at follow-up. This was identified according to either a self-reported history of diabetes diagnosis, taking of anti-diabetic medicine after the baseline examination, or being found to have an FPG level of ≥7.0 mmol/L (126 mg/dL) at any of the periodic examinations. The date of the diagnosis (incidence) was defined as the examination visit date when a new case of diabetes was identified; otherwise follow-up was censored if participants remained nondiabetic at the last follow-up.

### Statistical analysis

We used SAS version 9.4 (SAS Institute, Cary, NC, USA) for our analyses. An exploration cohort (49,325 persons) that accounted for two-thirds of the cohort was selected randomly to develop the risk scores for predicting the incidence of diabetes. A Cox proportional hazards model was conducted in a stepwise manner, with candidate variables with a significance of *P *≤ 0.2 included in the initial model; then, variables with a significance of *P *> 0.05 were removed. We took no account of the interaction terms between the independent variables. We refer to this model, which includes only demographic and anthropometric variables, as the concise model. The concise model supplemented with the laboratory evaluations results in the accurate model. For each model, the hazard ratio and 95% CI were calculated to estimate relative risk. In addition, β-coefficients were calculated to assign points for each risk factor by dividing the sum of the β-coefficients from the two models by 2 and rounding to the nearest integer. Continuous variables included in the model were categorized so that the estimated contribution of these factors to diabetes risk could be expressed through simplified point scores assigned to each of categories^13^. The sum of these points for each model was further calculated to predict the hazard of incidence of diabetes over a follow-up period of a mean of 5.35 years for each person.

ROC curves were used to compare the predictive discrimination of different risk scores. Additionally, the AUC (also referred to as C statistic) was used to give a quantitative assessment of the predictive ability of the score. Sensitivity and specificity were used to differentiate the subjects who developed diabetes from those who did not. A cut-off value was identified based on the optimal point that gave the maximum sum of sensitivity and specificity.

Our literature reviewed 40 original articles (dated from March 2000 to December 2013) that developed new diabetes risk scores. These included 20 articles that aimed to screen individuals with undiagnosed diabetes or impaired glucose tolerance and 20 articles that aimed to identify individuals at high risk of developing diabetes during a certain period. Among the 20 articles identifying the risk of diabetes incidence, we selected 11 articles^4^,^6^,^7^,^8^,^9^,^10^,^11^,^12^,^13^,^14^,^15^ for validation using our cohort (according to better AUC, and information scores available in the Kailuan study and different territories). The article by Griffin *et al*.^18^ was selected for its development of the Cambridge Diabetes Risk Score, which has proven to be effective in identifying those at risk for incident diabetes^42^; for the same reason, we also selected the article by Kanaya *et al*.^19^. We also tested the New Chinese Diabetes Risk Score^17^, which was originally developed to detect undiagnosed diabetes.

All validations were analysed using a 10-fold cross-validation method. The concise and accurate scores were validated in one-third of the cohort (24,662 participants). The other algorithms from different countries were also separately validated in the validation cohort and the whole cohort. We divided the validation cohort or the whole cohort into 10 smaller samples and validated 9 of them each time. We repeated the cross-validation process 10 times, and then calculated the mean AUC of the 10 validating values for the AUCs.

## Additional Information

**How to cite this article**: Wang, A. *et al*. Risk scores for predicting incidence of type 2 diabetes in the Chinese population: the Kailuan prospective study. *Sci. Rep.* **6**, 26548; doi: 10.1038/srep26548 (2016).

## Figures and Tables

**Table 1:** The baseline characteristics of the exploration and validation cohorts.

Characteristic	Exploration cohort (2/3)			Validation cohort (1/3)			*P*‡
	New diabetes (*N *= 4,726)	No diabetes (*N *= 44,599)	P*	New diabetes (*N *= 2,327)	No diabetes (*N *= 22,335)	*P*†	
Age (years)	52.38 ± 10.46	49.48 ± 12.16	<0.001	52.34 ± 10.47	49.48 ± 12.15	<0.001	0.95
Gender (Men)	3,959 (83.77%)	34,937 (78.34%)	<0.001	1,940 (83.37%)	17,493 (78.32%)	<0.001	0.85
Marital status							
Married	4,692 (99.28%)	43,705 (98.00%)	<0.001	2,314 (99.44%)	21,896 (98.03%)	<0.001	0.64
Other	34 (0.72%)	894 (2.00%)		13 (0.56%)	439 (1.97%)		
Income level (¥)							
<800	4,125 (87.28%)	37,948 (85.09%)	<0.001	2,013 (86.51%)	19,064 (85.35%)	0.03	0.23
800–1,000	336 (7.11%)	3,601 (8.07%)		184 (7.91%)	1,704 (7.63%)		
≥1,000	265 (5.61%)	3,050 (6.84%)		130 (5.59%)	1,567 (7.02%)		
Sleep duration (h)	7.29 ± 2.35	7.30 ± 4.42	0.77	7.23 ± 2.14	7.27 ± 4.78	0.15	0.06
Height (cm)	167.67 ± 6.87	167.47 ± 7.01	0.04	167.49 ± 6.93	167.48 ± 7.02	0.71	0.87
Weight (kg)	74.48 ± 11.57	69.69 ± 11.22	<0.001	74.32 ± 11.38	69.66 ± 11.24	<0.001	0.78
Waist-to-hip ratio	0.91 ± 0.06	0.89 ± 0.07	<0.001	0.91 ± 0.06	0.89 ± 0.07	<0.001	0.13
Waist circumference, men/women (cm)							
<84/77	945 (20.00%)	15,147 (33.96%)	<0.001	464 (19.94%)	7,649 (34.25%)	<0.001	0.76
84–89.9/77–83.9	1,096 (23.19%)	12,003 (26.91%)		546 (23.46%)	5,978 (26.77%)		
≥90/84	2,685 (56.81%)	17,449 (39.12%)		1,317 (56.60%)	8,780 (38.99%)		
BMI (kg/m^2^)							
<24.0	1,122 (23.74%)	18,952 (42.49%)	<0.001	561 (24.11%)	9,491 (42.49%)	<0.001	0.93
24.0–28.0	2,134 (45.15%)	18,301 (41.03%)		1,068 (45.90%)	9,163 (41.03%)		
≥28.0	1,470 (31.10%)	7,346 (16.47%)		598 (30.00%)	3,681 (16.48%)		
Physical activity frequency							
Never	471 (9.97%)	4,045 (9.07%)	<0.01	230 (9.88%)	2,122 (9.50%)	<0.001	0.08
Occasionally	3,482 (73.68%)	33,865 (75.93%)		1,676 (72.02%)	16,816 (75.29%)		
Frequently	773 (16.36%)	6,689 (15.00%)		421 (18.09%)	3,397 (15.21%)		
Salt intake							
Low	439 (9.30%)	4,141 (9.29%)	0.08	212 (9.13%)	2,117 (9.48%)	0.16	0.36
Medium	3,719 (78.76%)	35,586 (79.83%)		1,822 (78.43%)	17,718 (79.38%)		
High	564 (11.94%)	4,849 (10.88%)		289 (12.44%)	2,486 (11.14%)		
Smoking habit							
Never	2,723 (57.65%)	26,340 (59.14%)	<0.01	1,298 (55.88%)	13,181 (59.06%)	0.01	0.46
Ex-smoker	260 (5.50%)	2,299 (5.16%)		127 (5.47%)	1,220 (5.47%)		
Occasional	143 (3.03%)	1,712 (3.84%)		87 (3.75%)	853 (3.82%)		
Frequent	1,597 (33.81%)	14,190 (31.86%)		811 (34.91%)	7,064 (31.65%)		
Drinking							
Never	2,662 (56.35%)	25,751 (57.78%)	<0.001	1,288 (55.40%)	12,702 (56.90%)	<0.001	0.14
Ex-drinker	176 3.73%)	1,458 (3.27%)		87 (3.74%)	746 (3.34%)		
Occasional	854 (18.08%)	9,289 (20.84%)		440 (18.92%)	4,771 (21.37%)		
Frequent	1,032 (21.85%)	8,067 (18.10%)		510 (21.94%)	4,103 (18.38%)		
Daily sedentary time (h)							
<4	3,594 (76.05%)	33,059 (74.12%)	<0.01	1,764 (75.81%)	16,554 (74.12%)	0.12	0.36
4–8	974 (20.61%)	10,125 (22.70%)		497 (21.36%)	5,013 (22.44)		
≥8	158 (3.34%)	1,415 (3.17%)		66 (2.84%)	768 (3.44%)		
Family history of diabetes	307 (6.50%)	1,929 (4.33%)	<0.001	156 (6.70%)	998 (4.47%)	<0.001	0.37
CVD§	179 (3.79%)	1,081 (2.42%)	<0.001	91 (3.91%)	536 (2.40%)	<0.001	0.92
Using lipid-lowering drugs	52 (1.10%)	316 (0.71%)	<0.01	28 (1.20%)	181 (0.81%)	0.04	0.14
Education (high school or lower)	4,530 (95.85%)	40,988 (91.90%)	<0.001	2,210 (94.97%)	20,621 (92.33%)	<0.001	0.16
Blood pressure (mmHg)							
SBP < 120 or DBP < 80	1,214 (25.69%)	17,742 (39.78%)	<0.001	599 (25.74%)	8,916 (39.92%)	<0.001	0.09
120 ≤ SBP < 140 or 80 ≤ DBP < 90	2,162 (45.75%)	19,260 (43.18%)		1,079 (46.37%)	9,754 (43.67%)		
SBP ≥ 140 or DBP ≥ 90 or using anti-hypertensive drugs	1,350 (28.57%)	7,597 (17.03%)		649 (27.89%)	3,665 (16.41%)		
Resting heart rate (bpm)							
60–69	1,179 (24.95%)	13,610 (30.52%)	<0.001	633 (27.20%)	6,789 (31.40%)	<0.001	0.83
70–89	3,135 (66.34%)	28,393 (63.66%)		1,477 (63.47%)	14,238 (63.75%)		
≥90	412 (8.72%)	2,596 (5.82%)		217 (9.33%)	1,308 (5.86%)		
FPG (mmol/L)							
<5.6	2,073 (43.86%)	36,116 (80.98%)	<0.001	1,025 (44.05%)	18,115 (81.11%)	<0.001	0.54
5.6–6.1	1,132 (23.95%)	5,759 (12.91%)		537 (23.08%)	2,922 (13.08%)		
6.1–6.9	1,521 (32.18%)	2,724 (6.11%)		765 (32.87%)	1,298 (5.81%)		
TG ≥ 1.70 mmol/L	2,164 (45.79%)	12,896 (28.92%)	<0.001	1,058 (45.47%)	6,468 (28.96%)	<0.001	0.96
TC ≥ 5.72 mmol/L	1,306 (27.63%)	9,123 (20.46%)	<0.001	596 (25.61%)	4,692 (21.01%)	<0.001	0.35
HDL < 1.03 mmol/L (men) HDL < 1.29 mmol/L (women)	416 (8.80%)	4,193 (9.40%)	0.18	220 (9.45%)	2,032 (9.10%)	0.57	0.35
LDL ≥ 2.59 mmol/L	1,873 (39.63%)	16,365 (36.69%)	<0.001	910 (39.11%)	8,307 (37.19%)	0.07	0.29

Key: BMI – body mass index; CVD – cerebrovascular diseases; SBP – systolic blood pressure; DBP – diastolic blood pressure; FPG – fasting plasma glucose; TG – Triglyceride; TC – total cholesterol; HDL – high density lipoprotein; LDL – low density lipoprotein.

^*^Significance level for the difference between new diabetes and no diabetes in the exploration cohort.

^†^Significance level for the difference between new diabetes and no diabetes in the validation cohort.

^‡^Significance level for the difference between the exploration and validation cohorts.

^§^CVD is defined as fatal and nonfatal myocardial infarction, ischaemic stroke, or haemorrhagic stroke.

**Table 2:** Coefficients and HRs (95% CI) of models and values of risk scores for predicting incident diabetes in the exploration cohort using the Cox proportional hazards model.

	Concise score		Accurate score		Score
	HR (95% CI)	Coefficient	HR (95% CI)	Coefficient	
Age (years)					
18–29	Ref.		Ref.		0
30–39	2.03 (1.61–2.56)	0.71	1.74 (1.38–2.18)	0.55	6
40–49	3.26 (2.63–4.05)	1.18	2.66 (2.15–3.30)	0.98	10
50–59	3.20 (2.58–3.96)	1.16	2.59 (2.09–3.21)	0.95	10
60–69	3.28 (2.62–4.10)	1.19	2.90 (2.32–3.62)	1.06	11
≥70	3.16 (2.48–4.03)	1.15	2.94 (2.30–3.75)	1.08	11
Gender (Men)	1.34 (1.23–1.44)	0.29	1.14 (1.05–1.23)	0.13	2
BMI (kg/m^2^)					
<24.0	Ref.		Ref.		0
24.0–28.0	1.68 (1.56–1.81)	0.52	1.44 (1.33–1.55)	0.36	4
≥28.0	2.68 (2.48–2.91)	0.99	2.08 (1.91–2.25)	0.73	9
Family history of diabetes	1.66 (1.48–1.86)	0.51	1.45 (1.29–1.64)	0.37	4
Education (high school or below)	1.34 (1.16–1.55)	0.29	1.38 (1.20–1.60)	0.32	3
Blood pressure (mmHg)					
SBP < 120 or DBP < 80	Ref.		Ref.		0
120 ≤ SBP < 140 or 80 ≤ DBP < 90	1.25 (1.14–1.38)	0.22	1.17 (1.06–1.29)	0.16	2
SBP ≥ 140 or DBP ≥ 90 or using anti-hypertensive drugs	1.62 (1.47–1.78)	0.48	1.37 (1.24–1.51)	0.31	4
Resting heart rate (bpm)					
<70	Ref.		Ref.		0
70–79	1.16 (1.08–1.25)	0.15	1.07 (0.99–1.15)	0.07	1
80–89	1.36 (1.25–1.49)	0.31	1.15 (1.06–1.25)	0.14	2
≥90	1.77 (1.58–1.98)	0.57	1.26 (1.13–1.42)	0.23	4
FPG (mmol/L)					
<5.6			Ref.		0
5.6–6.1			2.94 (2.73–3.16)	1.08	11
6.1–6.9			7.05 (6.59–7.55)	1.95	20
TG ≥ 1.70 mmol/L or using lipid-lowering drugs			1.41 (1.33–1.49)	0.34	3
AUC of the risk scores	0.67		0.77		

Key: BMI – body mass index; SBP – systolic blood pressure; DBP – diastolic blood pressure; FPG – fasting plasma glucose; TG – Triglyceride; AUC – area under the receiver operating characteristic curve.

**Table 3:** The diagnostic characteristics of the concise and accurate scores in predicting diabetes in the validation cohort.

	No. of participants	Concise score				Accurate score			
		AUC	Cut-off	Sensitivity	Specificity	AUC	Cut-off	Sensitivity	Specificity
Total	24,662	0.66 (0.65–0.68)	21	0.72	0.52	0.77 (0.76–0.78)	27	0.70	0.70
Men	19,433	0.65 (0.63–0.66)	21	0.66	0.55	0.76 (0.74–0.77)	33	0.65	0.73
Women	5,229	0.72 (0.70–0.75)	17	0.61	0.71	0.81 (0.78–0.83)	20	0.70	0.76
Age < 60	20,275	0.67 (0.66–0.68)	17	0.70	0.55	0.76 (0.75–0.78)	25	0.72	0.68
Age ≥ 60	4,387	0.62 (0.59–0.64)	20	0.65	0.52	0.77 (0.75–0.79)	29	0.77	0.64

Key: AUC – area under the receiver operating characteristic curve.

**Table 4:** The performance of other risk scores in predicting incident diabetes and detecting undiagnosed diabetes in our cohort.

Year	Leading author	Population	Risk factors	Contains laboratory variables?	Validating cohort (1/3) AUC (95% CI)	Full cohort AUC (95% CI)
2000	Griffin	English	Age, sex, prescribed antihypertensive medication, BMI, *first degree relative had diabetes, non-smoker, ex-smoker, current smoker, (prescribed steroids).	No	0.60 (0.58–0.61)	0.60 (0.59–0.61)
2002	Stern	Mexican American, and non-Hispanic whites	†Age, sex, ethnicity, FPG, SBP, HDL, BMI, family history of diabetes.	Yes	0.71 (0.70–0.73)	0.71 (0.71–0.72)
2003	Lindström	Finnish	‡Age, BMI, waist circumference, use of blood pressure medication, §history of high blood glucose.	No	0.58 (0.56–0.60)	0.57 (0.56–0.58)
2005	Kanaya	Californian American	Age, sex, TG, FPG.	Yes	0.67 (0.66–0.69)	0.67 (0.66–0.68)
2005	Schmidt	American	||Age, black, parental history of diabetes, FPG, SBP, waist circumference, height, HDL, TG.	Yes	0.74 (0.73–0.76)	0.74 (0.74–0.75)
2006	Aekplakorn	Thai	¶Age, sex, BMI, waist circumference, hypertension, history of diabetes in parent or sibling.	No	0.60 (0.59–0.62)	0.60 (0.59–0.61)
2007	Schulze	German	Age, waist circumference, height, moderate alcohol, former smoker, current heavy smoker. (red meat, whole-grain bread, coffee, #physical activity)	No	0.59 (0.57–0.60)	0.58 (0.57–0.59)
2007	Wilson	White and non-Hispanic American	**BMI, parental history of diabetes mellitus, blood pressure >130/85 mm Hg or receiving therapy, HDL, TG, FPG.	Yes	0.53 (0.51–0.54)	0.52 (0.51–0.53)
2008	Balkau	French	††Men: waist circumference, current smoker, hypertension. Women: waist circumference, diabetes in the family, hypertension.	No	0.58 (0.57–0. 60)	0.58 (0.57–0.58)
2009	Chien	Chinese	Age, BMI, WBC, TG, HDL, FPG.	Yes	0.73 (0.71–0.74)	0.73 (0.72–0.74)
2009	Gao	Indian	‡‡Age, sex, BMI, waist circumference, FPG, TG.	Yes	0.73 (0.71–0.74)	0.72 (0.71–0.73)
2009	Kahn	American	##Age 55–64 y, diabetic mother, diabetic father, hypertension, black race, never drank alcohol or former drinker, waist circumference, height, resting pulse, FPG, TG, HDL, UA.	Yes	0.72 (0.70–0.73)	0.71 (0.71–0.72)
2010	Chen	Australian	Age, sex, BMI, race, waist circumference, parental history of diabetes, history of high blood glucose, use of antihypertensive medications, current smoker, physical inactivity.	No	0.59 (0.58–0.61)	0.59 (0.59–0.60)
2013	Zhou	Chinese	Age, sex, BMI, waist circumference, SBP, family history of diabetes.	No	0.61 (0.59–0.62)	0.61 (0.59–0.62)

The variables in parentheses were removed from the original model when validated because they could not be provided or be provided in sufficient detail in the Kailuan study. Key: BMI – body mass index; FPG – fasting plasma glucose; SBP – systolic blood pressure; HDL – high density lipoprotein; TG – Triglyceride; WBC –white blood cell; UA–uric acid; AUC – area under the receiver operating characteristic curve.

^*^In the original article, “parents or siblings had diabetes” was assigned 0.728 and 0.753, respectively. However, we did not discriminate between parents and siblings when assessing family history of diabetes in the questionnaire. We used the point sum for 0.728 and 0.753 and divided by 2 for this variable when validating.

^†^We selected the clinical model no 2 h glucose for validation from the 4 models in the original article.

^‡^We selected the concise model for validation because the variables included in it are provided in the Kailuan study and because it had a relatively good AUC in the original article.

^§^The variable ‘history of high blood glucose’ was replaced by ‘history of diabetes’ in the Kailuan study.

^||^We selected the model consisting of clinical variables plus fasting glucose and lipids, which had the highest AUC in the original article.

^¶^We selected the simple model from the 7 models described in the original article because it was defined with points and was recommended by the article.

^#^Physical activity in the original model was calculated per hour, which cannot be derived in such detail in the present study. It was removed when validating.

^**^We selected the simple clinical model in the original article because it was transformed into a point score, performed well with a good AUC, and was recommended in the article.

^††^We selected the clinical risk score in the original article because it was defined with points and the variables in the other model cannot be obtained for the Kailuan study.

^‡‡^We selected the accurate model in the original article because it performed better with a better AUC.

^##^We selected the enhanced diabetes prediction model, which had a higher AUC than the basic model in the original article.
